# Christmas in the Institutions

**Published:** 1915-01-02

**Authors:** 


### CHRISTMAS IN THE INSTITUTIONS.

### Christmas at Addenbrooke's, Cambridge.

### HOW IT IS PREPARED FOR AND HOW IT IS SPENT.

The advent of Christmas at Addenbrooke's is heralded by the preparation and making of the plum-puddings. This important but preliminary portion of the preparations commences usually in November, so that these puddings of old fame are naturally well matured by the time they are required for use.

### The Festivities Fund.

Quite early in December the Secretary-Superintendent, Mr. R. J. Coles, commences his annual appeal for contributions towards the special fund for providing the customary festivities at Christmas for the patients and staff. There are always those who for some reason or other are unwilling to give contributions of money to such a fund, but the Secretary-Superintendent at Addenbrooke's is equally unwilling to allow these friends to escape their obligations, and so solicits their assistance in the form of gifts in kind, to which there is a ready response.

It is a curious fact, but nevertheless true, that in all appeals it is prudent to take care that the contribution list is headed with a substantial sum to give every encouragement to those who at heart are anxious to contribute liberally, but who so often by reason of being oversensitive are unwilling to give any sum which is likely to exceed in amount those already given. Great care is taken at Addenbrooke's that no weakness of this kind shall find a place. The Secretary-Superintendent sees to it that the list is headed with a substantial donation from some friend of the hospital. The members of the general committee are invited to contribute to the fund usually at their first meeting in December. The Secretary-Superintendent then appeals systematically to a large number of friends of the institution, as well as to tradesmen, for contributions of money and gifts in kind. The appeal is made both by personal letters and through the medium of the columns of the local papers.

Some ten years ago the fund for Christmas festivities seldom reached a higher figure than about £7, and the gifts in kind were of a very limited character. Last year the contributions to this fund in money amounted to £37, and in addition substantial gifts in kind.

### The Critic of Christmas.

The critic, with his eagle eye, is ever ready to complain of waste, luxury, and the appropriation of funds for what he considers improper purposes. Conscious of the susceptibilities of the critic, care has been taken at Addenbrooke's Hospital, Cambridge, that no such attack can be made. The cost of the whole of the Christmas festivities is defrayed out of the fund specially raised for the purpose. This year the fund amounts to about £31, or a little less than the amount received last year, as is only to be expected having regard to the many additional demands made upon the generosity of friends of the institution. Upon the other hand, the gifts in kind, such as turkeys, fowls, game, potted meat, fruit, vegetables, cakes, sweets, jellies, jams, articles of warm clothing for men, women and children, toys, etc., flowers and evergreens, have been substantially more than in past years.

### How the Feast is Kept.

By Christmas Eve the ward sisters and their nurses have their respective wards decorated and illuminated in a most artistic and appropriate manner.

The Halton Ward (women's medical) was decorated by the sister, Miss Sendall, and her nurses in a very pleasing manner—the entrance to the ward with evergreens, flags, and fairy lamps. The electric-lamp shades were covered with very pretty yellow paper shades, the bed-pulls with evergreens and yellow festoons. Yellow paper festoons were linked in one continuous chain from one bed-pull to another on both sides of the ward. From each bed-pull was suspended a Japanese lantern of a colour blending with the general decorations. The white columns over the central fireplaces were entwined with evergreens and yellow paper festoons. In the centre of the columns, suspended from the ceiling, was an electric light covered with a very pretty Chinese lantern, and the base of the columns was profusely decorated with choice flowers. In like manner the ward table exhibited great taste in the way in which it had been set out with palms, plants, and flowers. On each of the window sills were to be seen flowers and fairy lights.

In the Victoria Ward (women's surgical)—the largest in the hospital—the sister, Miss Somerville, and her nurses displayed great taste in carrying out the decorations. Paper shades, pink and purple in colour, covered the electric-light shades. The bed-pulls were decorated with evergreens and pink and purple fancy paper, and from each bed-pull hung a lantern of a soft green colour. Here, again, on the window sills, tables, etc., was to be seen a display of very choice flowers, plants, and fairy lights. The columns over central fireplaces were entwined with trails of ivy, etc., and on the side facing the entrance of the ward a trophy of flags of the allied nations.

The Humphry, or Children's, Ward, under the charge of Miss Somerville, was equally well decorated, as also was the corridor connecting the two wards. A Christmas tree, in height about 10 feet, was to be seen illuminated by means of electric lights, thereby minimising any risk of danger from fire, and laden with suitable gifts for the children, which were distributed on Boxing Day by "Father Christmas" (Captain Mallam, R.A.M.C., acting house surgeon).

The new ward on the top of the hospital, recently completed and as yet officially unnamed (but for the present known as "Tipperary"), is being used temporarily as a combined male medical and surgical ward in consequence of the usual male medical and surgical wards being closed for the remodelling of the bath annexe attached. Miss Swan, the sister, together with her nurses, employed the colours red, yellow, and black. The electric-lamp shades, both of the pendants, and the brackets over each bed were very prettily bedecked with yellow fancy paper shades in the form of a flower. On the ward table there was a display of choice flowers, ferns, etc. Here, again, fairy lamps in a variety of colours were used, thus producing a combination of illumination.

### The Male Surgical Ward.

Albert Ward (male surgical). This ward, which for a time was closed during the erection of the new ward immediately over, and to enable the bath annexe attached to be remodelled, has been completed and repainted throughout. Miss Tully, the sister, and her nurses, full of zeal and enthusiasm, produced an ultimate effect which moved everyone to admiration. On approaching the entrance to the ward there was a rustic arch richly decorated with clinging evergreens and fairy lights which at night produced a very pretty effect. The colours adopted for the general decorations were scarlet and green. Over the shades of the electric pendants were fixed very artistic scarlet fancy paper shades entwined with clinging evergreens. The bed-pull brackets over each bed were decorated with evergreens, and at the end of each bracket was fixed a large scarlet bow of fancy paper, and there were very pretty Japanese lanterns suspended from each bed-pull bracket. The window sills were bedecked with evergreens, fairy lamps, and vases of fine flowers in beautiful colours. The ward tables were also decorated with singular taste.

The Hope and Collignon Wards.

These wards were very daintily decorated in red, yellow, and black colours at this time familiar. The bed pulls were linked up with a continuous chain of evergreen and fancy paper festoons; the table with evergreens, plants, and flowers; the electric-lamp pendants with fancy paper shades in yellow and black, and from some there hung a large butterfly. All the window sills and recesses were bedecked with evergreens, plants, and choice fairy lights. At night when fully illuminated a most pleasing effect was to be seen. Miss Keilher, nurse in charge, her probationers, and Miss Barrance, who assisted them in their very difficult task, deserve much credit.

On Christmas Eve the matron, sisters, and nurses processed through the darkened wards, carrying Japanese lanterns on sticks, and singing carols, which the patients thoroughly enjoyed.

On Christmas morning all the patients aroused from their slumbers to find that Santa Claus had even paid them his accustomed visit, leaving for each a parcel. During the morning the patients' friends were allowed to visit them from 9 to 10.30 A.M.

The Christmas Spread.

At 12 o'clock noon the usual Christmas dinner took place, at which function the house physician, Captain Budd, R.A.M.C., dressed as a *chef*, carved in the new "Tipperary" Ward; Captain Mallam, R.A.M.C., house surgeon, dressed as a *chef*, carved in Victoria Ward; Alderman John Chivers carved in Halton Ward; the Matron carved in Hope and Collignon Wards; and the Secretary-Superintendent, dressed as "John Bull," carved turkey in Albert Ward, which the patients subsequently annexed.

At 1.30 the maids had their Christmas dinner, the matron and doctors carving and the sisters waiting. After tea for the patients, at 3.30 the members of the nursing staff as a troupe, under title "Kitchener's Last Resource," gave a patriotic concert in each ward.

On Boxing Day, presided over by the matron, at 8 P.M. the nursing staff sat down to their Christmas dinner, when Captain Budd, house physician, and 18 old Addenbrooke's nurses now on duty at 1st Eastern General Hospital were also present. The tables were decorated exquisitely in shades of pink and with pink carnations. A small concert in Albert and the new wards brings the festivities this Christmas-tide to a close. Other years there have been concerts in each ward, but this year such have been curtailed, as was only to be expected having regard to the present circumstances. The patients have thoroughly enjoyed the season, and it is worthy of note that not one is the worse for the excitement.

### Great Northern Central Hospital.

HIS MAJESTY'S GIFT.

UNDER the present circumstances Christmas at the Great Northern Central Hospital could not be spent quite in the usual joyous way, but the committee, staff, and friends of the institution were united in their efforts to make the season for the patients and wounded soldiers bright and happy.

Each ward was tastefully decorated by the sisters and nurses, the national colours of Great Britain and the Allies being strongly in evidence. On Christmas morning carols were sung, and at 12 o'clock an excellent dinner was served in each of the wards, consisting of roast turkey, with the usual adjuncts, plum-pudding, and mince-pies, followed by dessert and bonbons. There were few who were not able to enjoy at least some of the good things provided, and friends were allowed to visit the patients between 2 and 4 in the afternoon.

On Boxing Day a special Christmas tea was given in each ward by the Ladies' Association of the hospital, when suitable presents were distributed to every patient, including the wounded soldiers, also music and songs were enjoyed on this occasion. The children's ward had its usual Christmas tree, the kind gift of Lord Islington, loaded with toys and presents, and the small inmates in the cots knew nothing but delight when they gazed on the glittering lights, and national troubles were for the time almost forgotten in watching their pleasure. The committee were fortunate in receiving a large number of seasonable gifts for the patients, including a large box of toys from the Queen for the children. The festivities terminated with an excellent entertainment graciously provided by his Majesty the King.

### Fulham Infirmary.

Durum! Sed levius fit patientia,
Quidquid corrigere est nefas.

THE lines of Horace, last read in class, with glimpses of blue skies and green boughs through the high narrow windows of the room, and long forgotten, came back fresh to the lips by some freak of memory. Christmas Day: a grey, cold, misty morning; the ordered wards bright with decorations as one has known them on so many past Christmases, and yet with a subtle difference hardly realised at first, but soon apparent. A woman, dressed in black and older than her years, pauses suddenly as she leaves the patient whom she has come to see—pauses because two lads in khaki are passing her to the next bed. Impulsively she puts her hand upon the arm of the nearer one. "Excuse me," she says, as she presses his hand and raises it for a moment to her lips, "God bless you! I have two—wounded—in Netley." Indeed "it is hard, and yet patience makes it easier to bear what it were dishonour to change." Here is the keynote to the thoughts of all and to the form which the celebration of the day takes. The war has entered into our lives. None but has seen one of his own step proudly from the open gates of the temple of Janus to pass along the road at the end of which the choosers are hovering. Our thoughts dwell the more upon that far off birthday when for a brief space the gates clanged to and the whole world was at peace; but the gaiety, the lightness of heart of past years are not for us to-day.

"I wish you a happy Christmas, I cannot wish you a merry one"—this greeting of one of the ward sisters expresses well the distinction we feel. The effect is seen in all the wards—not so much in the flags which wave everywhere, in those which are chosen and those which are eschewed, not even in the absence of the old phrases—"On earth, peace" is not for us, and even "good will toward men" cannot find a place—as in the general restraint in the decorations and the merry-making.

Only the children are, happily, unconscious of the change. Like the children of past years they fell asleep on Christmas Eve while asserting their determination "only to pretend" so that they might see "Father Christmas" come, the more so that the absence of chimneys to the ward stoves puzzled them as to his mode of access as it has puzzled their predecessors. And the pleasure of the well-filled stocking on their awakening drove away their disappointment at their failure. For them the full flavour of the Christmas fare, the delight of visiting the other wards and comparing the decorations with their own, the pleasure of the afternoon concerts, the ecstasy of the lighted Christmas trees in the evening, and the sweetness of the carols sung by nurses and choristers in procession through the wards. They did not notice that the excellent song of the Herald Angels gave place to the quieter carols speaking of trials and hardships for which a new hope and a solution, at variance with all previous conception, had been born into the world. They did not notice, or noting did not realise, the significance of the Belgian refugee fingering the red, yellow, and black drapery at one of the ward entrances, and murmuring proudly "My Flag." So Christmas passed—a Christmas such as we have never known in our generation, and, pray God, such as we and our children may never know again.

### Wilkinson Sanatorium, Bolton.

CHRISTMAS, with its festivities, is looked upon at the Wilkinson Sanatorium as a useful tonic to be made much of in the dull days, and this year its helpfulness has been considered more needed than usual; consequently a full programme has been carried out with the help of kind friends, the patients themselves also entering heartily into providing amusements. The decorations, which consisted of red art muslin, mottoes, Chinese lanterns and sunshades, and a "Father Christmas" in the hall to welcome you, with the help of a "first-class" concert, gave a good start, and roused keen interest as to what to expect.

When Christmas morning arrived carol singing "surrounded" breakfast time, and the usual routine gave way very largely to meeting friends during the day, the evening being devoted to games and the amusement of "fishing" in a dry but not empty pond for various little useful and fancy things provided by the generosity of a lady friend. This "fish pond" has an interesting side to a careful observer. The value of the little bag, shawl, blouse, or something suitable for "the wife," the pin tray, glove box, or "something that can be given to the best friend next time they meet" means much to those who have had no chance of shopping or giving pleasure for weeks past, and to those who are specially sensitive and grateful there is great pleasure in something to "keep" in memory of the day. The parcels are wrapped in quantities of paper according to the size of the article, so that a pencil parcel is very much the same size as a doll parcel. Consequently, it often happens that a man "fishes" the doll while a girl hooks the shaving-brush; then there is business done and exchange is made, unless the man is a father who wishes to keep the doll for his little girl. Possibly someone will fish a bell or rattle or something noisy; in fact, many a joke is fished out of the pond, and if there is anyone that the fun then doesn't touch—well, that one is a failure indeed.

The dinner-table was a dainty display of crackers on a red table centre surrounded with green, and fancy serviettes were provided for each one. The turkey was carved by one of the trustees, who acts as "operating surgeon" for this annual occasion, and all did justice to the ample supply of good things. For two Sundays the usual service has given place to special music. On December 20 members of St. Paul's Church choir, with their organist, gave a selection of delightful carols, and on December 27 Mr. Nightingale, the secretary of the sanatorium, presided, while selections from the "Messiah" were well rendered. The Old Year finished with a very amateur performance by the patients themselves of "The Village Wedding."

### Christmas in a Chinese Hospital.

IN the Church Missionary Society Hospital at Hangchow, though we are a very large "family," we have no definite way of keeping Christmas, but before it arrives all our people are busy preparing decorations which, when finished, make our hospital and compound very bright and cheerful. In our chapel last Christmas we had decorations almost entirely of green, chiefly holly, which was redundant in lovely berries. The Chinese gardeners and others are splendid in making all kinds of things—balls, stars, and various devices with pine, cypress, holly, etc. Around our garden, which is more or less public to the hospital "family," we hang red lamps at intervals which are lit at sundown. Last Christmas Dr. Strange took round a band of students to sing carols at the doors of the missionaries' houses, and the "girl-nurses" in the Women's Hospital ushered in Christmas by singing carols in English, which they had practised specially for the occasion.

The students in the Medical College usually get up some entertainment, and are very good in giving representations of Chinese life which, with singing and other entertainments, provide a very pleasant evening. We usually have a Christmas tree either in the Children's Home or Women's Hospital, and the women and children are invited to it, and we generally make up parcels for all on the compound, about 150, as either a Christmas or New Year gift, while there are special services in our hospital chapel to explain to our patients the real meaning of Christmas—a season which is a very happy time, for all our helpers enter into the spirit of happiness and good-will.

### Leeds Hospital for Women and Children.

THIS year the decorations are much more simple, but the wards look very pretty with paper lampshades and fresh flowers. Victoria Ward is decorated to represent autumn. The centre lampshades are made of bronze paper lined with pale pink, and trimmed with autumn leaves, and side-lights of pink tulips festooned with autumn leaves, and ivy festoons across the ward. The flowers are bronze chrysanthemums, pink carnations, and pink roses and trails of smilax.

Alexandra Ward is decorated to represent spring, with orange paper centre shades, and side-lights of daffodils. In this ward the flowers are white and yellow chrysanthemums, smilax, and white tulips. The centre table has a large white and green centrepiece, with flowers, candle shades of yellow, and fairy lights.

"Maternity" looks very pretty, with white and green Japanese shades, side-lights of pale yellow tulips, and the cots tied up with apricot bows. The babies also have the same coloured ribbons tying up their wrists.

At present the great attraction in the maternity wards are twin baby girls owning the names of "Mary" and "Jean," and a Cæsarean baby named "Bobs," after Lord Roberts. Each patient in the hospital received a present from Santa Claus, and the day nurses sang carols at an early hour, their voices sounding well along the corridors. At midday the patients had their Christmas dinner, consisting of turkey, plum pudding, and dessert; and during the afternoon each patient had a visitor to tea. In Victoria Ward the honorary surgeon provided the visitors with cigars and cigarettes, which were very much appreciated. The nursing and domestic staff had their dinners on Christmas Day. The nurses' dining-room was most patriotic, with the Allies' colours and flags.

The nurses are entertaining the patients to two sketches during the week, and they are giving daily concerts in the wards.

### Christmas in a Base Hospital.

Colonel Dr. Connell, the officer commanding the 3rd Northern General Hospital, Sheffield, received a convoy of 150 wounded two days before Christmas. Owing to the success of his scheme for sending wounded when they are well enough out to convalescent homes the base hospital was only about half full. On Christmas Day the thirty Belgian soldiers present apparently enjoyed themselves almost as much as the British "Tommies." The Bishop of Sheffield (Dr. Hedley Burrows) celebrated Holy Communion at the hospital at 7 a.m., and after breakfast Colonel Connell presented every man with a newly-minted shilling. After each man had received two presents there were still sufficient to permit of the R.A.M.C. men being given a couple each. For the Christmas dinner there were fifty-four turkeys sent, 800 plum-puddings of different sizes, 900 cakes, over 2,000 mince-pies, apples, oranges, dates, figs, bananas, and a host of other things. The men were regaled well again on Boxing Day. The afternoon was devoted to a popular variety concert, and in the evening there was an impromptu concert, items being given by the R.A.M.C. men, the nurses, and certain of the wounded. On the platform in the dining-hall stood a huge Christmas iced cake, weighing 200 lb., which had been given by a Sheffield lady named Mrs. Stevenson.

### The National Sanatorium, Benenden, Kent.

To learn that we had to go away for treatment at a sanatorium a few days before Christmas seemed to be the climax of a run of bad luck, but upon reflection, now that the festivities are over, we are inclined to think that "good luck" was with us after all.

We were roused early on Christmas morning by the crowing of a human cockerel in the West Wing, and by the answers of his feathered companions in the farmyard.

The sound of the night nurse's slippers along the corridor and into our cubicles with a nice warm mug of tea and biscuits quickly disabused us of misgivings we had had of rising on this "cold and frosty morning" without "something hot" to give us a good start. The shouts of the patients who had the temerity to hang up their stockings overnight were only eclipsed by the chorus of approval that was heard when our friend and patient, Mr. H. M. Stewart, gave us an imitation of Christmas bells on the concertina.

The skittle-alley claimed the attention of a number of patients after breakfast, whilst others went for a walk. Just before 1 p.m. the patients were marshalled at their places in the dining-hall for dinner. The hall had been decorated by the patients and staff with festoons of holly and bay-leaves together with an abundance of flags and bunting, and prominent in the decorations were admirable oil-paintings inscribed, "To give you greeting," from the Anerley Postal Staff.

Waited upon by the medical superintendent, Dr. Cox, the secretary-steward, Mr. H. Seagrave, and his wife, matron and nurses, and usual kitchen staff, dinner proceeded with unusual chaff and laughter. Having cleared the tables, the patients set out to amuse themselves during the remainder of the day.

During the afternoon a number of patients went for a walk, some played billiards, whilst others amused themselves by relating anecdotes in the dining-hall. After tea we had a visit from Mr. Newbold, a visitor at Cleveland's Farm, and his reminiscences of the late Dr. Parker were entertaining and instructive. It was a matter of regret when the *raconteur* had to leave us for his "regulation walk," and the patients were unanimous in their thanks to him for the witty anecdotes.

Left to our devices we quickly made arrangements for a smoking concert, and, with Mr. Dudley in the chair, a most enjoyable evening was spent. We should like to express our appreciation of the efforts of Mr. Heath and his admirable rendering of "The Night Watchman's Story" and a selection from Charles Dickens's "Only Way" (*sic*); Mr. H. M. Stewart for the excellent solos on the concertina; Mr. Dudley, Mr. Cooper, Mr. Clarke, and Dr. Cox for songs; and our thanks to Mr. G. F. Doran for his performance at the piano.

### Cardiff.

### KING EDWARD VII.'S HOSPITAL.

Each hospital has a special feature in its recognition of Christmas, and probably the special feature of King Edward VII.'s Hospital, Cardiff, is its Christmas tree. This is always placed in the Children's Ward—at present the beautiful "Coronation" Ward—and exactly at five o'clock on Christmas Eve the matron touches a button and the tree becomes dazzling with myriad electric lights, and then out steps Father Christmas and gives a present to everyone. Everything happened just as usual this year. At first one saw no difference from other years; yet there were differences, the wounded soldiers who came in from their part of the hospital to join in the children's festivity; many familiar faces were missing; and the khaki: certainly the ward had never seen so many military uniforms before, khaki blending with the blue of the wounded soldiers and a large sprinkling of Territorial nurses. These were the obvious differences that suggested the war, but within and around there was apparently peace and such happiness as loving thought and bright kind hearts can always help to make. Excellent concerts followed the Christmas tree in both the military and civil sections of the hospital.

Christmas Day had its usual routine, visits to the wards by kind and generous friends, the good cheer presided over by the patients' own doctors. How they all enjoyed the turkeys and the plum-puddings and the seasonable fare generally. Then concerts again in different wards, and there will be a series of concerts all through the week up to the New Year. How much of encouragement the patients give themselves. Here is an extract from a letter written to the Secretary, Mr. L. D. Rea, on Boxing Day by a patient who was able to leave just before Christmas: "May the Great God

Who guides the destiny of all things guard and further the efforts of those who are responsible for the carrying on of such great and noble work as is being carried on at such places as King Edward VII.'s Hospital, Cardiff." To that heartfelt prayer we may say, in all sincerity and humbleness, "Amen"?

### The Cardiff Military Hospitals.

Christmas Day at Cardiff was essentially one for the wounded and sick soldiers lying in the military hospitals, and everything possible was done to make their lot a happy one. At the head of the organising arrangements were Colonel Hepburn (in command of the 3rd Western General Hospital) and Major Ewen Maclean (adjutant and registrar), who visited each section. Bounteous fare and seasonable gifts were distributed by a bevy of ladies. Donors of all sorts of "goodies" were represented, from the King and Queen down to humble citizens. The wards had been splendidly decorated, thanks to the head of the Territorial Force Nursing Association, Mrs. Trevor Tyler, and the chief of the enthusiastic body of auxiliary workers, Mrs. Ambrose, of Llandaff. These ladies have also been responsible for the mufti refitting of some 2,000 soldiers. The Christmas dinner consisted of many good things, and the nursing staff sacrificed their usual dinner hour in order to wait upon them. In the afternoon visitors were permitted to see the soldiers, and in the evening there were concerts by well-known musical parties belonging to the city. The new Lord Mayor, Alderman J. T. Richards, accompanied by the Lady Mayoress and their eldest daughter, went the round of the hospitals and distributed pipes, cigarettes, and tobacco to the wounded.

### Gravesend Hospital: A Hostile Aeroplane.

The presence of English wounded in the wards as well as representatives of our Belgian and French Allies made Christmas especially interesting. It was, however, essentially a children's festival, for in the children's ward nothing had been curtailed—the glittering and toy-laden Christmas-tree and Santa Claus (impersonated by the house surgeon) were both there, but in the other wards no lavish scheme of decoration or of set entertainment had been entered upon. There was seasonable fare and sweets and delicacies in abundance, and the music and cinematograph pictures which kind friends provided were greatly appreciated and much enjoyed.

Each man from the Expeditionary Force received a Christmas card from the King and Queen—photographs with facsimile autographs; a box of cigarettes from H.M. Queen Alexandra, and a box of chocolates. Another nice feature was a gift of two turkeys from money provided by officers on one of the "silent watchers" on the North Sea.

An unusual, and decidedly unwelcome, visitor in the shape of a hostile aeroplane which flew over the hospital about 1.15, and which was smartly chased by a British biplane, created no little interest and excitement. The hum of the motors and the booming of big guns attracted both patients and staff to the windows, from which the exciting chase could be seen for several minutes. As the machines were lost in the distance, the boom and thud of anti-aircraft "twelve-pounders" told us that fort after fort were taking their part in a most exciting flying fight down the Thames. There was much speculation during the afternoon as to whether the intruder had been brought down. We sincerely hoped so.

